# Psychometric properties of the Spanish version of the media health literacy questionnaire (MeHLit-SV)

**DOI:** 10.3389/fpubh.2024.1440386

**Published:** 2024-09-24

**Authors:** Noelia Navas-Echazarreta, Raúl Juárez-Vela, Antonio Martínez-Sabater, Vicente Gea-Caballero, Elena Chover-Sierra, Emmanuel Echaniz-Serrano, Regina Ruiz de Viñaspre-Hernández, Blanca Jodrá-Esteban, Pilar Sánchez-Conde, María Teresa Fernández-Rodrigo, Antonio Rodríguez-Calvo, Pedro José Satústegui-Dordá

**Affiliations:** ^1^Doctoral Program in Health Sciences and Sports, University of Zaragoza, Zaragoza, Aragon, Spain; ^2^Predoctoral Researcher in Training, University of La Rioja and the Autonomous Community of La Rioja, Logroño, Spain; ^3^Faculty of Health Sciences, University of La Rioja, Logroño, La Rioja, Spain; ^4^GRUPAC Research Group, Department of Nursing, University of La Rioja, Logroño, La Rioja, Spain; ^5^Faculty of Nursing, Nursing Care and Education Research Group (GRIECE), GIUV2019-456, Nursing Department, University of Valencia, Valencia, Spain; ^6^Care Research Group (INCLIVA), Hospital Clínico Universitario de Valencia, Valencia, Spain; ^7^Faculty of Health Sciences, Research Group Community Health and Care, Valencian International University, Valencia, Spain; ^8^Internal Medicine Department, Hospital General Universitario, Valencia, Spain; ^9^SAPIENF (B53_23R) Research Group, Department of Physiatry and Nursing, Faculty of Health Sciences, University of Zaragoza, Zaragoza, Spain; ^10^Media Literacy in Health Group (GRUPAMES)—Innovation and Training in Educational Sciences Research Center (CIFICE), University of Zaragoza, Zaragoza, Spain; ^11^Hospital Universitario de Salamanca, Salamanca, Spain; ^12^Faculty of Medicine, University of Salamanca, Salamanca, Spain

**Keywords:** media literacy, media health literacy, cross-cultural adaptation, psychometric validation, questionnaire

## Abstract

**Introduction:**

Media health literacy emerges as a response to the vast array of informational disorders prevalent in media communications. Given the absence of a measurement tool for this type of literacy in Spanish-speaking communities, the aim of the present study is to conduct a cross-cultural adaptation of the Media Health Literacy (MeHLit) questionnaire into Spanish and to analyze its psychometric properties in a sample of nursing students.

**Methods:**

The Spanish version of the MeHLit questionnaire (MeHLit-SV) was obtained through a process involving translation, back-translation, evaluation of the proposed items by a group of 22 experts, and a pilot study with 80 Spanish nursing students. Content validity was assessed using each item’s content validity index (CVI) and Aiken’s V (VdA), while internal consistency was evaluated through Cronbach’s Alpha.

**Results:**

Following the translation and adaptation process, the final version of the MeHLit-SV comprised 21 items organized into five dimensions. The CVI values exceeded 0.82 for all items, and the overall content validity index (S-CVI) was 0.9. Furthermore, the results of Aiken’s V surpassed the threshold considered acceptable (0.70). After piloting, the questionnaire demonstrated high internal consistency with a Cronbach’s alpha value of 0.936.

**Conclusion:**

The findings of this research support the reliability and validity of the MeHLit-SV for use among nursing students to measure their level of media health literacy. This questionnaire, with satisfactory psychometric properties and ease of administration, is an useful tool for assessing whether individuals possess the necessary skills to accurately analyze health information they encounter on a daily basis.

## 1 Introduction

Media health literacy is a key component in health promotion and self-care improvement. Digital media contribute to the spread of misinformation due to their characteristics, including immediacy, widespread content dissemination, and the impossibility of complete verification (1–5). One of the topics of greatest interest to citizens is health-related information. Therefore, individuals are exposed to the risk of acquiring information containing misinformation that may harm their health, leading them to modify their care habits without relying on scientific evidence (6).

In this context, acquiring knowledge to comprehend, evaluate, and effectively utilize health information becomes essential for making informed decisions about health (7, 8). In this regard, media health literacy is defined as the acquisition and promotion of skills or abilities that prioritize critical thinking in the analysis of health messages. This type of literacy emerges as a response to the vast array of misinformation prevalent on various mass media platforms today (9). Consequently, individuals with higher levels of literacy can adopt a critical and active stance toward the health information they encounter and identify those of questionable quality (10–12).

At the international level, both the European Union and UNESCO emphasize the critical importance of media literacy education. Since 2014, Europe has integrated media literacy into its educational framework through targeted programs (13). In Spain, however, the approach has been less structured. While recognized as a transversal competence, media literacy has neither been established as a formal subject nor given clear evaluation criteria in the Spanish education system.

This lack of formal integration, combined with inadequate teacher training, underscores the urgent need for effective improvements in an era characterized by information overload and systemic disinformation (14–16). Despite efforts by Spanish educational institutions to foster critical reading and digital competence, and universities’ focus on media literacy research, the outcomes have fallen short of expectations (14).

Furthermore, collaborative initiatives between the Spanish Ministry of Education and various Autonomous Communities have failed to yield significant progress. This is particularly evident in Andalusia, a region in southern Spain, where numerous projects aimed at enhancing media competencies have been implemented since 2013. However, these efforts have not translated into measurable improvements in citizens’ media literacy (16–18).

When focusing on population health, achieving an adequate level of media health literacy poses multiple challenges, as this process is influenced by socio-economic, educational, and cultural factors. These factors affect individuals’ capacity to access, comprehend, and effectively utilize health information. Disparities in access to healthcare, understanding of health and illness concepts, or cultural health practices can significantly impact health outcomes and individuals’ ability to make informed decisions about their well-being (11, 19).

Determining the level of media health literacy among different population groups serves as an effective tool for assessing the risks to their self-care practices and, consequently, their overall well-being (19). Therefore, the development of a reliable media health literacy measurement scale adapted to the Spanish population is crucial in the current context of disinformation. This instrument can provide an assessment of the critical analysis skills of the target population and highlight the need to adopt a critical attitude toward health-related information disseminated in the media (20). Media health literacy can be quantified through the various indicators and characteristics that define it (6).

Levin-Zamir et al. (11), through the Media Health Literacy (MHL) scale, merged two complementary concepts: media literacy and health literacy, combining in a single scale the capacity to analyze media and health information and utilize it effectively. In constructing their scale, Levin-Zamir et al. (11) focused on understanding the message content, both explicitly and implicitly. Additionally, their dimensions centered on the perceived effect on individual behavior, the capacity for critical analysis, and the willingness to adopt new habits when a message is perceived as beneficial for health.

The adaptation of the Media Health Literacy (MeHLit) questionnaire of Nazarnia et al. (21) to Spanish not only involves the literal translation of items but also considers cultural and linguistic differences that may influence the understanding and interpretation of health information. Perception of health and illness, media consumption habits, and cultural beliefs can impact how individuals interpret, comprehend, and utilize health information (7, 11, 12). These factors influence the level of media health literacy in the population and the modification of their healthy habits (12).

According to a study by Sádaba Chalezquer et al. (22), media literacy has historically been a focal point in Communication Faculties for teaching and research. Nevertheless, its interdisciplinary nature allows for its application in diverse fields. With the emergence of the COVID-19 pandemic, ensuring citizens possess adequate media health literacy has become crucial, given the prevalence of health-related misinformation. Furthermore, amidst the current challenge of media disinformation, it is essential for all healthcare professionals to strengthen their authority as health experts and enhance their symbolic capital (23, 24).

The competencies of nursing professionals emphasize the significance of health promotion and effective communication in enhancing the quality of life and healthcare delivery (25, 26). Introducing media literacy in health into the university education curriculum for nursing students is essential for enhancing their training and improving the quality of healthcare they will provide in their future careers. It is crucial to assess the level of health media literacy among students as a target population, and by diagnosing their initial situation and implementing effective improvement measures, future nurses can develop strategies to empower citizens in making informed health decisions (27, 28).

The present research aims to address this crucial health need by providing a measurement instrument in Spanish. Accordingly, the objective of this study was to adapt the Media Health Literacy (MeHLit) questionnaire by Nazarnia et al. (21) to the Spanish language and analyze its psychometric properties in a sample of nursing students. The current context underscores the importance of media health literacy, as well as justifying its transcultural adaptation. Furthermore, the study represents a significant step toward improving health communication and combating misinformation by reaching out to the Spanish-speaking population.

## 2 Materials and methods

### 2.1 Study design

**Figure 1:**
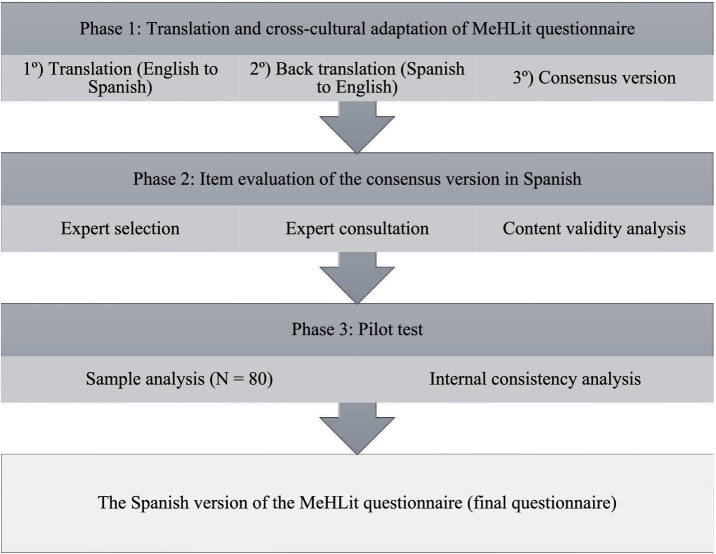
Diagram of the phases in the validation study of the Spanish version of the Media Health Literacy (MeHLit) Questionnaire.

The current study focused on adapting the MeHLit scale into Spanish for cross-cultural use and assessing its psychometric properties in a group of nursing students. The research was carried out from February to March 2024, following a three-phase methodology (see Figure 1) as recommended by the International Test Commission (29) guidelines for test adaptation.

Initially, the MeHLit scale was translated and culturally adapted from English to Spanish. Subsequently, a group of experts evaluated the proposed items. Finally, a pilot test was conducted with a sample of Nursing Degree students from the University of Zaragoza, who belonged to the target population for the Spanish version of the current scale (29, 30). The pilot test involved a self-developed questionnaire that gathered sociodemographic data from the students and their feedback on the understanding of the items. Prior to participating in the pilot test, students were briefed on the research and consented to take part.

### 2.2 Media health literacy questionnaire

The Media Health Literacy (MeHLit) questionnaire developed by Nazarnia et al. (21) offers a scale that effectively measures individuals’ ability to comprehend health messages with validity and reliability. However, its availability solely in English creates significant barriers for its usage in Spanish-speaking communities. Recently, there has been a cross-cultural adaptation to the Chinese language among adult populations, as conducted by Li et al. (31), demonstrating satisfactory psychometric properties.

The current study has undertaken the cross-cultural adaptation of the MeHLit questionnaire by Nazarnia et al. (21) into Spanish. This questionnaire, comprising 21 items and 5 dimensions, evaluates the skills and abilities associated with media health literacy, namely goal appraisal skill (seven items), content appraisal skill (five items), implicit meaning appraisal skill (four items), visual comprehension skill (three items), and audience appraisal skill (two items), with scores ranging from 0 to 84. Higher scores reflect a better understanding of health-related messages in the media. The questionnaire exhibits high internal consistency, evaluated through Cronbach’s Alpha at 0.91 and content validity with a Content Validity Index (CVI) of 0.93, based on a sample of 213 adults.

In the case of its transcultural adaptation to the Chinese language (31), this questionnaire proved to be an advantageous tool for evaluating the level of media health literacy among adults, with high internal consistency values (Cronbach’s alpha = 0.85; McDonald’s omega = 0.83).

### 2.3 Adaptation process

First, permission to proceed with the adaptation process was obtained from Dr. Fatemeh Zarei, the author of the MeHLit scale. Upon receiving consent, an initial version of the scale was created by translating it from its original English version (21) into Spanish.

The translation process then involved three bilingual translators with expertise in media health literacy research and proficiency in both Spanish and English as indicated by Hambleton and Lee (32) and Squires et al. (33). Each translator independently produced a Spanish translation of the questionnaire, resulting in three translated versions of the MeHLit (MeHLit-1, MeHLit-2, and MeHLit-3). Subsequently, a fourth translator, proficient in both languages, compared the three translations with the original scale to identify potential ambiguities and discrepancies in expression, phrases, and meanings.

In the event of discrepancies among the three translations, the fourth translator discussed these differences with the three translators via video conferencing to reach an agreement. An expert committee, consisting of three professionals with relevant doctorates and extensive experience in nursing research, journalism, health literacy, and instrument development, was established to address cases where an agreement could not be reached. Through consensus, the committee resolved inadequate expressions and harmonized the translations, ultimately merging the three versions into a consensus Spanish version (MeHLit-4).

The back-translation process (34) of the Spanish version of MeHLit-4 into English was then carried out by two bilingual translators with specialized training in English linguistics, who had not previously accessed the original English version. This procedure resulted in two independent back-translated versions, labeled MeHLit-5 and MeHLit-6. To ensure the accuracy and coherence of the back-translations, the expert committee and the five participating translators conducted a comprehensive comparative analysis between the back-translated versions and the original English scale.

### 2.4 Selection of experts

Prior to determining the final version of the scale in Spanish, contact was made with 22 experts (29, 30). This group consisted of healthcare professionals, journalists, advertising and public relations experts, as well as digital content creators with extensive experience in social media. All experts had a minimum of 3 years of professional experience in their respective fields. The group of 22 experts comprised 12 healthcare professionals with nursing qualifications (three of whom were content creators), five advertisers (one of whom was a content creator), and five journalists (one of whom was a content creator).

Among the 12 healthcare professionals, three were engaged solely in clinical practice, six supplemented their clinical work with teaching and research at the university, and the remaining three were exclusively dedicated to teaching and research at the university.

The experts were contacted via email to invite them to participate in the transcultural adaptation. This email presented the study’s objective and the characteristics of their participation, including the criteria for expert selection, the voluntary nature of participation, the confidential and anonymous data recording, and instructions for completing the survey. The survey was sent to them in the same email in the form of a Microsoft Forms platform questionnaire. It gathered their opinions on the adequacy, comprehension, and relevance of the items in the MeHLit scale translated into Spanish. Prior to completing the survey, interested experts had to accept the written consent provided, which adhered to the Organic Law 3/2018, of December 5, on the Protection of Personal Data and Guarantee of Digital Rights, applicable in Spain.

### 2.5 Expert panel consultation

The experts participating in the study were asked to evaluate the wording, comprehensibility, and relevance of each item (29, 30) in the provisional Spanish version of the MeHLit scale. In this initial phase, experts assessed these three properties of each item using a four-point Likert scale, where 1 indicated “totally disagree” and 4 indicated “totally agree.” Additionally, a qualitative response question was included, allowing experts to provide their opinions and suggest improvements regarding the comprehension and clarity of each item. In the second phase, a discussion group was formed to consider the experts’ suggestions and subsequently modify the questionnaire, defining its final version.

### 2.6 Content validity analysis

The analysis of the questionnaire’s content validity was conducted by calculating the Content Validity Index (CVI) and Aiken’s V value for each item. A minimum threshold of 0.6 was established for both CVI and Aiken’s V value for item inclusion in the questionnaire, in accordance with the criterion adopted for element selection. These indicators were calculated based on evaluations provided by experts. Two content validity indicators were determined for each item, using the following equations and the ratings given by the panel of experts:

- Content Validity Index (CVI), according to Polit and Beck (35). The calculation of the Content Validity Index (I-CVI) for each item was performed individually, using the ratings provided by the panel of experts, following the formula:
$$CVI=\;\frac{\mathrm{Number of experts}\;\mathrm{who}\;\mathrm{evaluated the item with}\;3\;\mathrm{or}\;4}{\mathrm{Total of experts}\;\left(N\right)}$$

Then, the global questionnaire content validity index (S-CVI), defined as the arithmetic mean of the I-CVI was obtained. CVI values that are equal to or greater than 0.78 are considered acceptable, whereas values equal to or greater than 0.90 are considered indicative of high content validity (35).

- V de Aiken according to the equation proposed by Penfield & Giacobbi (36):
$$V=\frac{X-l}{k}$$

Where *X* denotes the average of the experts’ assessments, *l* represents the minimum attainable score, and *k* signifies the span of feasible values within the utilized Likert scale.

Following the calculation, confidence intervals for Aiken’s V were determined using the scoring method (37).

To derive the lower limit of this interval, the following equation was utilized:

$$L=\frac{2nkV+z^{2}-z\;\sqrt[{}]{4nkV\left(1-V\right)+z^{2}}}{2\left(nk+z\right)}$$

For the upper limit:

$$U=\frac{2nkV+z^{2}+z\;\sqrt[{}]{4nkV\left(1-V\right)+z^{2}}}{2\left(nk+z\right)}$$

*L:* denotes the lower limit of the interval, *U:* represents the upper limit of the interval, *Z*: stands for the value in the standard normal distribution, V: signifies Aiken’s V calculated by formula 1, and *n:* denotes the number of experts.

### 2.7 Comprehensibility analysis

The comprehension validity was assessed based on expert evaluations of the level of understanding for each item. Items with an average score above 3 were considered highly comprehensible, those scoring between 2.5 and 3 were deemed moderately comprehensible, and items scoring below 2.5 were classified as having low comprehensibility.

The panel of experts offered suggestions to enhance the comprehensibility of the items, particularly focusing on those with lower scores. Moreover, during the pilot test, students were asked to evaluate the understandability of the proposed items.

### 2.8 Internal consistency analysis

The preliminary investigation (pilot test) allowed for an assessment of the internal consistency of the questionnaire through the calculation of Cronbach’s alpha. A threshold of 0.7 for Cronbach’s alpha is considered the minimum acceptable value, with scores below this indicating insufficient internal consistency of the instrument (38).

### 2.9 Statistical analysis

A database was constructed from an Excel 2013 spreadsheet to compute the CVI and Aiken’s V, following their respective formulas and utilizing the ratings provided by the experts. For the remaining statistical analyses, the SPSS program was employed (IBM SPSS Statistics for Windows, Version 28.0. IBM Corp., Armonk, NY, United States).

This included conducting a descriptive study of the variables utilized in the pilot test and calculating the internal consistency of the Spanish version of MeHLit.

### 2.10 Ethical considerations

The present study was conducted with the approval of the Ethics Committee of the University of La Rioja, under verification code (CSV) osoTEUuvlSV4cyZA9TJxqqVmc5motYSk, as documented in the link provided by the institution (University of La Rioja, 2024): *https://sede.unirioja.es/csv/code/osoTEUuvlSV4cyZA9TJxqqVmc5motYSk.*

## 3 Results

### 3.1 Results of the translation process

During the translation process from English to Spanish, certain concerns arose about the MeHLit questionnaire that required clarification. Among these was the necessity to precisely define the concept of implicit and hidden meaning in health media information. Specifically, in item 15, there was uncertainty about whether the information always inherently contains implicit or hidden meanings, or if instead, respondents should be asked about their capability to identify the implicit or hidden meaning of the information. To resolve this uncertainty, advice was sought from a journalist and the original scale’s authors. All concurred that information consistently encompasses both implicit and explicit meanings; hence, the phrase “I am able to identify its implicit and hidden meaning” was adopted.

### 3.2 Consensus MeHLit-SV

Table 1 displays the items of the MeHLit-SV consensus version provided to the experts, along with their English translations.

**Table 1:** Spanish version of MeHLit (MeHLit-SV).

Item number	MeHLit-SV item
1	Cuando leo, veo o escucho un mensaje relacionado con la salud, trato de averiguar cuál es su objetivo.
	When I read, watch, or listen to a health-related message, I try to find out what its purpose is.
2	Cuando leo, veo o escucho un mensaje relacionado con la salud, me planteo cuál es su finalidad educativa.
	When I read, watch, or listen to a health-related message, I consider its educational purpose.
3	Cuando leo, veo o escucho un mensaje relacionado con la salud, soy capaz de identificar y comprender su significado explícito y directo.
	When I read, watch, or listen to a health-related message, I am able to identify and comprehend its explicit and direct meaning.
4	Cuando leo, veo o escucho un mensaje relacionado con la salud, reflexiono sobre el significado que tiene para mí.
	When I read, watch, or listen to a health-related message, I reflect on its meaning for me.
5	Cuando leo, veo o escucho un mensaje relacionado con la salud, lo analizo desde distintos aspectos como su posible aplicación, utilidad o efectividad.
	When I read, watch, or listen to a health-related message, I analyze it from various aspects such as its potential application, usefulness, or effectiveness.
6	Cuando leo, veo o escucho un mensaje relacionado con la salud, valoro la posibilidad de borrarlo, conservarlo o compartirlo con otras personas.
	When I read, watch, or listen to a health-related message, I consider the possibility of deleting it, keeping it, or sharing it with others.
7	Cuando leo, veo o escucho un mensaje relacionado con la salud, valoro qué pensamientos e ideas promueve.
	When I read, watch, or listen to a health-related message, I assess the thoughts and ideas it promotes.
8	Cuando leo, veo o escucho un mensaje relacionado con la salud, me intereso por conocer cuál es la fuente de origen.
	When I read, watch, or listen to a health-related message, I am interested in knowing its original source.
9	Cuando leo, veo o escucho un mensaje relacionado con la salud, valoro quién lo publica.
	When I read, watch, or listen to a health-related message, I consider the publisher.
10	Cuando leo, veo o escucho un mensaje relacionado con la salud, me pregunto si todo el mundo lo entenderá de la misma manera.
	When I read, watch, or listen to a health-related message, I wonder if everyone will understand it in the same way.
11	Cuando leo, veo o escucho un mensaje relacionado con la salud, compruebo su exactitud.
	When I read, watch, or listen to a health-related message, I verify its accuracy.
12	Cuando leo, veo o escucho un mensaje relacionado con la salud, lo analizo de forma crítica.
	When I read, watch, or listen to a health-related message, I critically analyze it.
13	Cuando leo, veo o escucho un mensaje relacionado con la salud, valoro las consecuencias negativas y positivas de difundirlo.
	When I read, watch, or listen to a health-related message, I assess the negative and positive consequences of spreading it.
14	Cuando leo, veo o escucho un mensaje relacionado con la salud, valoro quién se puede beneficiar de que su contenido se difunda (beneficios económicos, de salud, sociales, etc.)
	When I read, watch, or listen to a health-related message, I consider who may benefit from its dissemination (economic, health, social benefits, etc.).
15	Cuando leo, veo o escucho un mensaje relacionado con la salud, soy capaz de identificar su significado implícito y oculto.
	When I read, watch, or listen to a health-related message, I am able to identify its implicit and hidden meaning.
16	Cuando leo, veo o escucho un mensaje relacionado con la salud, valoro quién o quiénes apoyan ese mensaje.
	When I read, watch, or listen to a health-related message, I assess who or what supports that message.
17	Cuando leo, veo o escucho un mensaje relacionado con la salud, soy capaz de identificar las técnicas empleadas para llamar la atención del público (efectos especiales como color, luz, sonido, etc.)
	When I read, watch, or listen to a health-related message, I am able to identify the techniques used to capture the public’s attention (special effects such as color, light, sound, etc.).
18	Cuando leo, veo o escucho un mensaje relacionado con la salud, valoro a través de qué medio se ha difundido (redes sociales, medios de comunicación, etc).
	When I read, watch, or listen to a health-related message, I consider through which medium it has been disseminated (social media, traditional media, etc.).
19	Cuando leo, veo o escucho un mensaje relacionado con la salud, me fijo en la fecha de publicación del mensaje.
	When I read, watch, or listen to a health-related message, I pay attention to the date of publication of the message.
20	Cuando leo, veo o escucho un mensaje relacionado con la salud, analizo a quién va dirigido.
	When I read, watch, or listen to a health-related message, I analyze the target audience.
21	Cuando leo, veo o escucho un mensaje relacionado con la salud, valoro si es beneficioso para mí o no lo es.
	When I read, watch, or listen to a health-related message, I assess whether it is beneficial for me or not.

This table shows the MeHLit-SV items in Spanish and their English translation (but not an English-validated version).

### 3.3 Content validity results

The 22 experts’ evaluations produced the CVI values and Aiken’s V test results for each item, detailed in Table 2. When analyzing the CVI, all items scored above 0.82, with the overall questionnaire CVI (S-CVI) reaching 0.9, well surpassing the acceptable threshold of 0.8 (39). Moreover, Aiken’s V results for each item were all satisfactory, exceeding the acceptable value of 0.70 (40).

**Table 2:** The content validity index (CVI) and Aiken’sV for each item of the Spanish version of MeHLit (MeHLit-SV).

Item number	CVI	S-CVI	Aiken’s V	IC CVI 95% (0.60–0.96)
1	0.82	0.90	0.74	(0.63–0.83)
2	0.90		0.85	(0.74–0.92)
3	0.77		0.74	(0.63–0.83)
4	0.86		0.79	(0.67–0.87)
5	0.90		0.80	(0.69–0.88)
6	0.86		0.86	(0.76–0.93)
7	0.95		0.88	(0.78–0.94)
8	0.95		0.88	(0.78–0.94)
9	1		0.90	(0.82–0.96)
10	0.90		0.82	(0.71–0.89)
11	0.82		0.74	(0.63–0.83)
12	0.90		0.90	(0.82–0.96)
13	0.95		0.94	(0.85–0.98)
14	0.86		0.83	(0.73–0.90)
15	0.82		0.73	(0.60–0.82)
16	0.95		0.85	(0.74–0.92)
17	0.86		0.77	(0.66–0.86)
18	0.95		0.88	(0.78–0.94)
19	0.90		0.85	(0.74–0.92)
20	1		0.91	(0.82–0.96)
21	0.95		0.91	(0.82–0.96)

CVI, Content validity index; IC, Confidence interval; S-CVI, Scale content validity index.

### 3.4 Comprehensibility analysis

The experts’ assessments of the items’ clarity were overwhelmingly positive. The experts’ suggested grammatical adjustments were predominantly focused on items 1, 2, and 3. Even though these items were rated as moderately to highly comprehensible, scoring below 3.5, they were still revised. Specifically, item 1, which attained a comprehensibility rating of 3.18, was modified, as well as item 2, which scored 3.31, and item 3, which scored 2.90. Furthermore, the experts’ most common recommendation was to change the initial wording of each item from “When I come across a health-related message” to “When I read, watch, or listen to a health-related message.”

Each of the research-involved experts verified their understanding of the final questionnaire, its concepts, and the corresponding item responses, expressing satisfaction with their appropriateness and clarity. Additionally, none of the experts indicated any questions or uncertainties while completing the questionnaire. Moreover, all participants in the pilot study outlined below confirmed their comprehension of the MeHLit-SV questionnaire’s items.

### 3.5 Pilot study results

A group of 80 nursing students from the University of Zaragoza completed a questionnaire. The survey included questions about their socio-demographic background, digital device usage, and the Spanish version of the Media Health Literacy questionnaire (MeHLit-SV), which went through a translation process and consultation with experts.

The average age of the students was 21 years, with 83.75% being female and 16.25% male. Around half of the students had previously attended public institutions (51.25%), while the others had studied in private (6.25%) or semi-private (42.5%) institutions, and the majority (76.25%) had received their education in an urban setting. Furthermore, 35% of the students were working while pursuing their studies. When it came to accessing media health information, 78.75% of the students in the pilot study preferred using smartphones.

The detailed results for the socio-demographic variables analyzed in the student sample are presented in Table 3.

**Table 3:** Sociodemographic characteristics.

Sociodemographic variables		Mean ± Sd	*N*	(%)
Age		20.9 ± 4.6		
Gender				
	Woman			
	Men		67	83.75
	Non-binary		13	16.25
Population nucleus				
	Rural		19	23.75
	Urban		61	76.25
Pre-university education institution				
	Public		41	51.25
	Private		5	6.25
	Charter		34	42.50
Employment				
	Yes		28	35.00
	No		52	65.00
The device through which you mainly acquire media health information				
	Smartphone		63	78.75
	Laptop		15	18.75
	Desktop computer		1	1.25
	Tablet		1	1.25

Sd, Standard deviation; %, percentage.

Regarding the results obtained after completing the Spanish version of the MeHLit questionnaire, the average score of the surveyed students was 60.8 points (Table 4).

**Table 4:** Descriptive statistics of the MeHLit-SV applied to the student sample.

Measures	MeHLit-SV score
M	60.8
Sd	13.23
Cv	0.14
Min	26
Max	79
*N*	80

M, Mean; Sd, Standard deviation; Cv, Coefficient of variation; Min, Minimum; Max, Maximum.

### 3.6 Internal consistency results

After the pilot test, using the responses from the participating students, Cronbach’s alpha was calculated. The Spanish version of the MeHLit questionnaire demonstrated high internal consistency with a Cronbach’s alpha value of 0.936.

Subsequently, the correlation between the items was analyzed, and it was observed that Cronbach’s alpha did not rise to a significantly higher value upon removing any of the items (Appendix 1).

## 4 Discussion

Media health literacy stands as a firm response to the current context of misinformation, and as a cornerstone in this endeavor, it must be evaluated. The Media Health Literacy Questionnaire (MeHLit) by Nazarnia et al. (21) is an instrument of high quality regarding its psychometric properties, with its translation into Chinese also yielding satisfactory results (31). Therefore, in the present research, it was decided to carry out its cross-cultural adaptation into Spanish and validate it in a sample of nursing students.

The main reason for choosing this population to study their level of knowledge regarding the critical analysis of health messages was their status as future healthcare professionals. Thus, in the near future, today’s students will provide care to patients who, in turn, will consume health-related information daily through the media (41, 42). In this regard, another aspect considered for its cross-cultural adaptation was the lack of a Spanish version of this questionnaire.

The Spanish version of the MeHLit has demonstrated adequate psychometric properties. Content validity yielded high results for all items, with a CVI well above desirable values. Additionally, the scale’s overall CVI stood at 0.9, similar to the one obtained in the Chinese version, which was 0.94 (31). Furthermore, an additional test was conducted to evaluate content validity, Aiken’s V, which reaffirmed the quality of this property with a value exceeding 0.70.

Regarding internal consistency, the MeHLit-SV also demonstrated satisfactory results (Cronbach’s Alpha = 0.936), surpassing the Cronbach’s alpha value of 0.85 obtained by Li et al. (31) and the English version by Nazarnia et al. (21), which was 0.91.

Both in the Chinese version and in the current Spanish version of the MeHLit, the 21 items composing the original scale (21) have been retained. In the MeHLit-SV scale, eliminating item 6 would only increase Cronbach’s alpha by three hundredths. This marginal increase in Cronbach’s alpha would not significantly impact the overall assessment, and it would entail removing a relevant question for assessing media health literacy, specifically, the reflection prior to information dissemination. This phase mitigates the mass and immediate spread of false or misinformation, promoting individuals’ critical analysis (43).

Thus, it can be affirmed that the Spanish version of the MeHLit possesses adequate validity and reliability, justifying the quality of this tool for measuring the level of media health literacy in adults, specifically in Nursing Degree students. According to the World Health Organization (44), possessing skills to critically analyze health-related information and identify the presence of misinformation represents progress in all areas, socially, medically, and academically. To achieve this, it is necessary to adapt strategies to the target population, considering their access to information, digital literacy, and health knowledge (44, 45).

Young people represent the population group most exposed to media messages, as they are the main consumers of content disseminated through social media and other digital channels. Information about health is disseminated on these channels, which may contain multiple instances of misinformation (46–48). However, Chang et al.’s study (49) highlighted the importance of digital education among older adults as well. Older adults increasingly rely on digital media to obtain health-related information, with media serving as a mediator between family health and the use of technological devices, especially smartphones.

While adequate health knowledge empowers citizens to make decisions that benefit their self-care, low levels of literacy pose a problem for both public and individual health (19). In this regard, the use of measurement scales for media health literacy, such as the MeHLit-SV, provides an opportunity to assess the risk to which different population groups are exposed. Thus, it is possible to contribute to the implementation of programs and actions that improve citizens’ knowledge and promote critical analysis of media information about health (12, 50).

## 5 Strengths and limitations of the study

Regarding the limitations of the study, the analysis of internal consistency was conducted through a pilot test with a sample of students from a Spanish university, thus requiring further multicenter studies with larger samples to evaluate the reliability of the MeHLit-SV in other contexts. Additionally, the fact that the participating university population had higher health knowledge may have influenced the mean score of media health literacy obtained. In this regard, it is recommended that subsequent intervention studies be conducted on more heterogeneous samples.

Similarly to the original version of the MeHLit, no cutoff points were established to classify individuals as adequately literate. Therefore, our future studies will focus on addressing these limitations.

## 6 Conclusion

The results of this research support the reliability and validity of the MeHLit-SV for use among nursing students to measure their level of media health literacy. This questionnaire, with adequate psychometric properties and easy administration, is useful for assessing whether individuals possess the necessary skills to correctly analyze the health information they consume daily. The MeHLit-SV can be used in any setting as it is a cross-cutting competency, especially in educational environments and clinical practice, administered to both patients and healthcare professionals.

This questionnaire enables the establishment of educational programs and public policies aimed at improving communication and health actions for the general population. Likewise, at the international level, having a Spanish version of the MeHLit promotes conducting larger-scale studies comparing the level of media health literacy in different contexts and validating it in other Spanish-speaking countries, where linguistic modifications of the scale may be minimal.

## 7 Implications for practice

The transcultural adaptation of the MeHLit into Spanish has significant implications for clinical practice and healthcare delivery in Spanish-speaking communities. This validated and culturally relevant tool can enhance communication between healthcare professionals and patients, enabling more effective and meaningful interaction. Healthcare professionals can use the MeHLit-SV to assess patients’ competency in media health literacy and tailor their communication and education strategies more precisely, thereby improving their understanding of information.

Moreover, the MeHLit-SV can be a useful tool in health promotion by providing a valid and reliable scale for assessing media health literacy. It also promotes the design of educational interventions tailored to the specific needs of the population being served, aimed at empowering individuals to critically analyze the health information they consume. These actions can enhance treatment adherence and informed decision-making, benefiting health self-care practices.

## Data Availability

The raw data supporting the conclusions of this article will be made available by the authors, without undue reservation.

## Appendix

Appendix 1 Item analysis of the Spanish version of Media Health Literacy (MeHLit) questionnaire.

**Table:**

Item	Correlation coefficient: item-total score	Cronbach’s alpha if item deleted
MeHLit-SV 1	0.690	0.932
MeHLit-SV 2	0.697	0.931
MeHLit-SV 3	0.557	0.933
MeHLit-SV 4	0.546	0.934
MeHLit-SV 5	0.582	0.932
MeHLit-SV 6	0.350	0.939
MeHLit-SV 7	0.600	0.933
MeHLit-SV 8	0.656	0.933
MeHLit-SV 9	0.695	0.932
MeHLit-SV 10	0.452	0.935
MeHLit-SV 11	0.706	0.930
MeHLit-SV 12	0.656	0.931
MeHLit-SV 13	0.488	0.933
MeHLit-SV 14	0.736	0.930
MeHLit-SV 15	0.793	0.930
MeHLit-SV 16	0.621	0.933
MeHLit-SV 17	0.573	0.933
MeHLit-SV 18	0.575	0.932
MeHLit-SV 19	0.481	0.936
MeHLit-SV 20	0.696	0.931
MeHLit-SV 21	0.627	0.933

MeHLit-SV, Spanish version of Media Health Literacy questionnaire.
